# Activation of Lipid Mediator Formation Due to Lipoprotein Apheresis

**DOI:** 10.3390/nu11020363

**Published:** 2019-02-09

**Authors:** Karsten-H. Weylandt, Christoph Schmöcker, Annika I. Ostermann, Laura Kutzner, Ina Willenberg, Stefanie Kiesler, Elisabeth Steinhagen-Thiessen, Nils Helge Schebb, Ursula Kassner

**Affiliations:** 1 Medical Department, Divisions of Hepatology, Gastroenterology, Oncology, Hematologyand Diabetes, Ruppiner Kliniken, Brandenburg Medical School, 16816 Neuruppin, Germany; christoph.schmoecker@mhb-fontane.de (C.S.); S.Kiesler@ruppiner-kliniken.de (S.K.); 2 Charité-Universitätsmedizin Berlin, corporate member of Freie Universität Berlin, Humboldt-Universität zu Berlin, Medical Department, Division of Hepatology and Gastroenterology (including Metabolic Diseases), Campus Virchow Klinikum, 13353 Berlin, Germany; elisabeth.Steinhagen-Thiessen@charite.de (E.S.-T.); ursula.Kassner@charite.de (U.K.); 3 Institute for Food Toxicology and Analytical Chemistry, University of Veterinary Medicine Hannover, 30173 Hannover, Germany; annika.ostermann@schebb-web.de (A.I.O.); l.kutzner@gmx.de (L.K.); ina.willenberg@gmail.com (I.W.); nils@schebb-web.de (N.H.S.); 4 Chair of Food Chemistry, Faculty of Mathematics and Natural Sciences, University of Wuppertal, 42119 Wuppertal, Germany

**Keywords:** lipid apheresis, hyperlipoproteinaemia, gas chromatography, oxylipins, lipid mediators, omega-3 polyunsaturated fatty acids, eicosapentaenoic acid, docosahexaenoic acid, lipidomics, LC-MS/MS, *n*-3 PUFA, *n*-6 PUFA

## Abstract

Lipoprotein apheresis reliably reduces low-density lipoprotein (LDL) cholesterol in patients with atherosclerotic disease and therapy-refractory hypercholesterolemia or elevated lipoprotein (a) (Lp(a)). Besides lowering lipoproteins and triglycerides, apheresis also decreases levels of essential omega-6 and omega-3 polyunsaturated fatty acids (*n*-6 and *n*-3 PUFAs) in blood plasma. In contrast, heparin-induced extracorporeal low-density lipoprotein precipitation (HELP) lipid apheresis might increase the formation of potentially pro-inflammatory and pro-thrombotic lipid mediators derived from *n*-6 and *n*-3 PUFAs. The study presented here analyzed lipid mediator profiles in the plasma of patients with hyperlipidemia treated by one of three different apheresis methods, either HELP, direct absorption (DA), or membrane filtration (MDF), in a direct pre- and post-apheresis comparison. Using gas chromatography and liquid chromatography tandem mass spectrometry (LC-MS/MS) we were able to analyze fatty acid composition and the formation of lipid mediators called oxylipins. Our data illustrate—particularly in HELP-treated patients—significant decreases of essential omega-6 and omega-3 polyunsaturated fatty acids in blood plasma but significant increases of PUFA-derived lipoxygenase-, as well as cyclooxygenase- and cytochrome P450-derived lipid mediators. Given that *n*-3 PUFAs in particular are presumed to be cardioprotective and *n*-3 PUFA-derived lipid mediators might limit inflammatory reactions, these data indicate that *n*-3 PUFA supplementation in the context of lipid apheresis treatment might have additional benefits through apheresis-triggered protective *n*-3 PUFA-derived lipid mediators.

## 1. Introduction

Atherosclerosis and cardiovascular events are major causes of morbidity and mortality in patients with severe hypercholesterolemia. Besides lifestyle risk factors such as smoking and obesity, familial hypercholesterolemia is one the major genetically determined metabolic risk factors [1,2,3]. Furthermore, lipoprotein (a) (Lp(a)) is an established independent cardiovascular risk factor [4].

For those patients who continue to develop clinically significant cardiovascular disease in spite of lipid-lowering medications, lipoprotein apheresis is an established therapeutic method to reduce low-density lipoprotein cholesterol (LDL-C) levels and Lp(a). Long-term lipoprotein apheresis treatment has been proven to reduce major cardiovascular events by more than 80% [5]. Besides lowering cholesterol, apheresis decreases oxidized LDL particles and fibrinogen levels [6,7,8,9]. Moreover, lipoprotein apheresis therapy is associated with increased expression of nitric oxide synthase in human endothelial cells, which exerts vasoprotective influences in patients [10,11]. Lipoprotein apheresis has also been shown to change plasma cytokine profiles and reduce inflammatory markers in patients with hypercholesterolemia and coronary heart disease [12].

Current apheresis methods operate with different physicochemical principles to clear the blood of lipoproteins. Precipitation is employed during heparin-induced extracorporeal low-density lipoprotein precipitation (HELP, Futura^®^), where separated plasma is mixed with an acetate-acetic acid buffer (pH at 4.85–5.15) and heparin. Heparin binds to LDL-C, very low-density lipoprotein cholesterol (VLDL), and Lp(a) and due to the acidic environment, together with fibrinogen forms insoluble precipitates which are then removed by a polycarbonate membrane. The cascade filtration or membrane differential filtration (MDF) system MONET utilizes a membrane that retains particles bigger than 1000 kDa through which the plasma is circulated after separation of the cellular parts of the blood.

Whereas HELP and MDF are plasma-based apheresis procedures, in low-density lipoprotein hemoperfusion and direct absorption of lipoproteins systems (e.g., DALI^®^ (DA)), whole blood circulates through an adsorber that contains polyacrylate-coated polyacrylamide beads. Elimination of lipoproteins such as LDL and Lp(a) occurs by binding of the positively charged APO B100 regions to the negative polyanions of the polyacrylamide surface.

Various studies have shown that omega-6 (*n*-6) and omega-3 (*n*-3) polyunsaturated fatty acids (PUFAs) and their mediators can directly influence the progression of inflammatory, cardiovascular, and autoimmune diseases [13,14]. The *n*-6 PUFA arachidonic acid (AA, 20:4 *n*-6) as well as the *n*-3 PUFAs eicosapentaenoic acid (EPA, 20:5 *n*-3) and docosahexaenoic acid (DHA, 22:6 *n*-3) can be processed by different enzymatic (e.g., cyclooxygenase, lipoxygenase, cytochrome P450 enzymes) and non-enzymatic pathways, resulting in a wide spectrum of highly active lipid mediators, so-called oxylipins [15].

Therefore, in addition to LDL cholesterol-lowering therapy, *n*-3 PUFAs such as docosahexaenoic acid (DHA, 22:6 *n*-3) and particularly eicosapentaenoic acid (EPA, 20:5 *n*-3) have been shown in the reduction of cardiovascular risk [16,17], and *n*-3 PUFA-derived lipid mediators might have cardioprotective effects [18]. However, in a pilot study with a small number of patients treated with heparin-induced extracorporeal low-density lipoprotein precipitation (HELP) apheresis, we found significantly decreased levels of plasma *n*-3 PUFAs as well as a trend towards an increase of 5- and 12-lipoxygenase (LOX) product formation [19]. Since the activation of lipoxygenases and arachidonic acid (AA, 20:4 *n*-6)-derived lipid mediators have been implicated in the development of inflammation and atherosclerosis [20], these observations might have consequences for the inflammatory state and cardiovascular risk associated with PUFA-derived lipid mediators in apheresis-treated patients.

Recently, we expanded our analyses to compare different lipoprotein apheresis methods. We showed significantly reduced PUFA concentrations in plasma due to apheresis using either heparin-induced extracorporeal low-density lipoprotein precipitation (HELP, Futura^®^) or low-density lipoprotein hemoperfusion and direct absorption of lipoproteins, DALI^®^ (DA). In contrast, apheresis using the membrane differential filtration (MDF) system MONET did not affect the amount of PUFAs in plasma [21].

The aim of the present study was to establish the effect of different lipoprotein apheresis methods (HELP, MDF, and DA) on *n*-6 PUFAs and *n*-3 PUFAs as well as the formation of their lipid mediator in plasma. By using gas chromatography (GC) for the analysis of fatty acids and liquid chromatography coupled to tandem mass-spectrometry (LC-MS/MS), we were able to show significantly increased levels of a variety of lipoxygenase (LOX)- as well as cyclooxygenase (COX)- and cytochrome P450 enzyme (CYP450)-derived lipid mediators either from *n*-6 or from *n*-3 PUFAs, in spite of the PUFA-lowering effect of HELP and DA apheresis.

## 2. Materials and Methods

### 2.1. Patients and Blood Sampling

As described in our previous studies [19,21], EDTA blood was sampled directly before an individual lipid apheresis session. Patients were allowed to eat before apheresis treatment. Apheresis procedures were performed using standard procedures and according to manufacturers’ instructions (HELP: Plasmat Futura; B.Braun, Melsungen, Germany; MDF and DA: MONET and DALI, respectively; Fresenius Medical Care, Bad Homburg, Germany). Plasma was obtained by centrifugation for 10 min at 4 °C and 1000∙*g*. Supernatants were aliquoted and kept at −80 °C until analysis of lipid metabolites by LC-MS/MS or GC.

For routine clinical chemistry analysis, samples from each patient were taken at the same time, and serum levels of total cholesterol, LDL-cholesterol, high-density lipoprotein cholesterol (HDL-C), and triglycerides were determined at the central routine laboratory facility of the Charité University Medicine in Berlin. The study was approved by the ethics committee of the Charité University Medicine Berlin in accordance with the Declaration of Helsinki.

### 2.2. Fatty Acid Measurements

Fatty acid methyl esters (FAMEs) were quantified by gas chromatography flame ionization detection (GC-FID) as described previously [22]. Briefly, lipids of 50 μL plasma of each sample were extracted with methyl-tert-butylether/methanol (MTBE/MeOH). Subsequently, hexane and methanolic HCl were added to the dried lipids extract and heated to 90–95 °C for 60 min. After cooling down, the content was then transferred to a 6% potassium carbonate solution and then centrifuged. Organic phase was removed and evaporated under a stream of nitrogen. The extract was finally reconstituted with MTBE/MeOH (9:1) for gas chromatography flame ionization detection.

### 2.3. Sample Preparation and LC/ESI-MS/MS

Extraction of lipid mediators from 500 µL human plasma was carried out as described [23,24]. In brief, the plasma samples (500 µL) were spiked with an internal standard solution. In order to extract the free oxylipins in serum, solid phase extraction (SPE) without subsequent protein precipitation was performed. In brief, SPE was carried out as follows: Oasis HLB-SPE-cartridges (3 mL, 60 mg, 30 μm particles; Waters, Eschborn, Germany) were cleaned with one column volume of ethyl acetate (EA) and one of methanol (MeOH). After the samples were loaded on preconditioned SPE columns, the columns were washed with two column volumes of 5/95 ACN/water acidified with 0.1% acetic acid. After drying the SPE column by applying low vacuum (0.2 bar), the analytes were eluted using 0.5 mL methanol followed by 1.5 mL ethyl acetate. The eluted samples were evaporated to dryness, reconstituted in 50 μL of methanol, and analyzed by LC-MS/MS as described [23]. Lipid mediators and deuterated standards used in this study were purchased from Biomol, Germany. Concentrations of lipid mediators are shown as pmol/L in plasma.

### 2.4. Statistical Analysis

Statistical analysis was performed using GraphPad Prism Software, Version 5.01 (La Jolla, CA, USA). Comparison was made using two-tailed paired Student’s *t*-test (for two groups) and one-way ANOVA (for three or more groups), assuming standard distribution. Post hoc analysis was performed by Bonferroni’s multiple comparisons test. The independence of categorical variables between the groups was analyzed by Fisher’s exact test. All values are presented as the mean ± SEM or as indicated. Statistical significance was set at *p* < 0.05.

## 3. Results

### 3.1. Patient Characteristics, Standard Lipid Parameters, and PUFA-Levels in Blood Plasma

**Figure 1:**
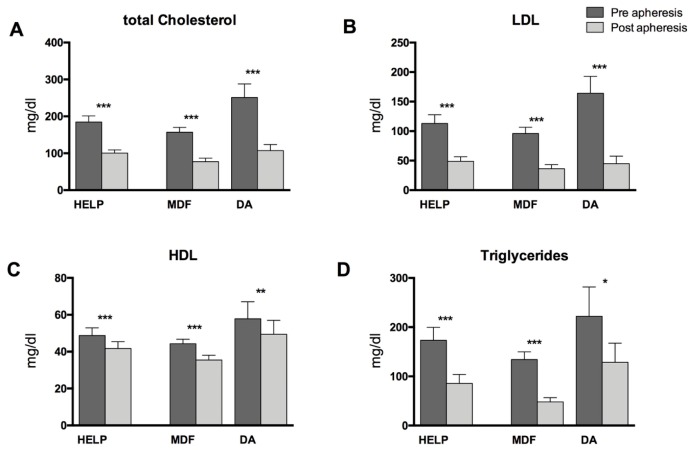
Routine lipid parameters in patients undergoing HELP, MDF, or DA apheresis. (**A**) shows concentrations of total cholesterol, (**B**) concentrations of low-density lipoprotein (LDL) cholesterol, (**C**) concentrations of high-density lipoprotein (HDL) cholesterol and (**D**) concentrations of triglycerides in the serum. Differences within one treatment group between pre- and post-lipoprotein apheresis were significant where indicated (* *p* < 0.05, ** *p* < 0.01, *** *p* < 0.001). Analysis was performed by two-tailed paired Student’s *t*-test.

Overall, samples from 17 patients undergoing HELP apheresis, from nine patients undergoing MDF, and from seven patients undergoing DA were analyzed (Table 1). Lipoprotein apheresis reduced total cholesterol and LDL-C by 50% and 63%, respectively, in the pre/post analysis. These results were accompanied by a mean decrease of 15.9% for high-density lipoprotein cholesterol (HDL-C) and a 51% decrease for the concentrations of triglycerides (TGs). Changes were statistically significant (Figure 1) and similar to the data we reported previously [19,21]. In this sample set, as well as in the samples assayed in our previous study, there were significant decreases in PUFA concentrations due to HELP as well as DA apheresis, but no significant changes in PUFA concentrations due to MDF were observed in the pre- and post-apheresis analysis (Table 2 and Figure S1).

### 3.2. Lipid Mediators Analysis

Lipid mediators in plasma were analyzed in samples taken directly before and after lipoprotein apheresis. We first compared pre-apheresis values between the groups. Baseline levels pre-apheresis were shown to be significantly different for DHA (22:6 *n*-3), with higher levels in the DA group. Furthermore, the concentrations of AA (20:4 *n*-6) and dihomo-gamma-linolenic acid (DGLA, 20:3 *n*-6) pre-apheresis were significantly lower in the MDF group. In both cases, post hoc analysis showed significant differences for the pairwise comparison of MDF and DA. Remaining *n*-6 and *n*-3 PUFAs were not significantly different between the three groups. For oxylipins, we only found significant differences in the variances for the two dihydroxy-metabolites, 8,9-dihydroxyeicosatetraenoic acid (8,9-DiHETE) and 14,15-DiHETE, with lower concentrations in the HELP group pre-apheresis. Variances for the remaining analyzed oxylipins did not significantly differ between the apheresis groups pre-apheresis treatment.

**Figure 2:**
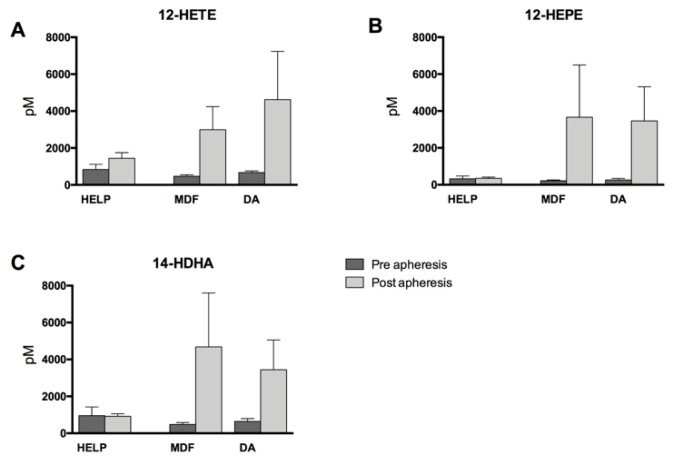
12-Lipoxygenase-derived monohydroxy lipid mediators directly before and after apheresis therapy. (**A**) Arachidonic acid (AA, 20:4 *n*-6)-derived 12-hydroxyeicosatetraenoic acid (12-HETE); (**B**) Eicosapentaenoic acid (EPA, 20:5 *n*-3)-derived 12-hydroxyeicosapentaenoic acid (12-HEPE); and (**C**) Docosahexaenoic acid (DHA, 22:6 *n*-3)-derived 14-hydroxydocosahexaenoic acid (14-HDHA). Differences within one treatment group pre- to post-apheresis were not significant for either HELP, MDF, or DA. Analysis was performed by two-tailed paired Student’s t-test. Differences between the treatment groups did not reach statistical significance as analyzed with one-way ANOVA.

Post-apheresis concentrations of 12-lipoxygenase (12-LOX)-derived metabolites (12-hydroxyeicosatetraenoic acid (12-HETE), 12-hydroxyeicosapentaenoic acid (12-HEPE), and 14-hydroxydocosahexaenoic acid (14-HDHA), derived from AA, EPA, and DHA, respectively) increased throughout, regardless of the apheresis method used. Indeed, the increase was least pronounced in the HELP-treated patients, while there were more pronounced increases of these mediators due to MDF or DA treatment (Figure 2). All increases within one treatment group and differences between the apheresis methods were not significant, however.

**Figure 3:**
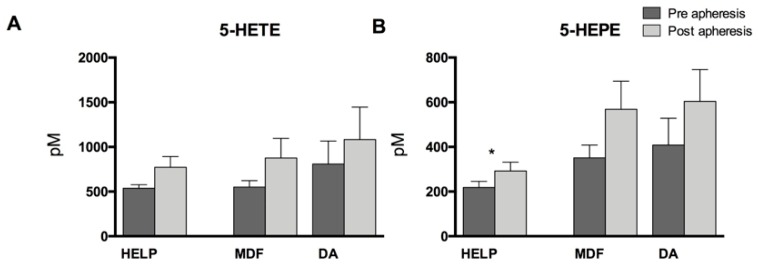
5-Lipoxygenase-derived (**A**) AA metabolite 5-hydroxyeicosatetraenoic acid (5-HETE) and (**B**) EPA metabolite 5-hydroxyeicosapentaenoic acid (5-HEPE). Differences within one treatment group between pre- and post-lipoprotein apheresis were significant where indicated (* *p* < 0.05). Analysis was performed by two-tailed paired Student’s *t*-test.

The AA and EPA metabolites of the 5-LOX pathway 5-hydroxyeicosatetraenoic acid (5-HETE) and 5-hydroxyeicosapentaenoic acid (5-HEPE) also increased due to apheresis, with a significant increase detectable in the HELP apheresis group (Figure 3).

**Figure 4:**
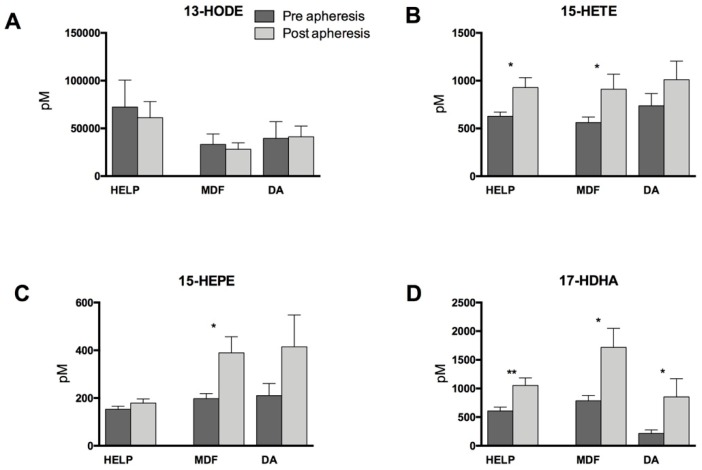
15-Lipoxygenase-derived monohydroxy lipid mediators directly before and after apheresis therapy. (**A**) Linoleic acid (LA, 18:2 *n*-6)-derived 13-hydroxyoctadecadienoic acid (13-HODE); (**B**) AA-derived 15-hydroxyeicosatetraenoic acid (15-HETE); (**C**) EPA-derived 15-hydroxyeicosapentaenoic acid (15-HEPE); and (**D**) DHA-derived 17-hydroxydocosahexaenoic acid (17-HDHA). Differences within one treatment group between pre- and post-lipoprotein apheresis were significant where indicated (* *p* < 0.05, ** *p* < 0.01). Analysis was performed by two-tailed paired Student’s *t*-test.

Interestingly, the linoleic acid- (LA, 18:3 *n*-6) and 15-LOX-derived metabolite 13-hydroxyoctadecadienoic acid (13-HODE) was slightly reduced by apheresis in the HELP and MDF groups. However, we found that lipoprotein apheresis significantly increased 15-LOX-derived mediators 15-hydroxyeicosatetraenoic acid (15-HETE), 15-hydroxyeicosapentaenoic acid (15-HEPE), and 17-hydroxydocosahexaenoic acid (17-HDHA) in the patient group treated with MDF apheresis. This finding is supported by significant changes in variances for 15-HEPE post apheresis treatment between the three different treatment groups. Increases of 17-HDHA were also significant in patients undergoing HELP or DA lipid apheresis, and 15-HETE also increased significantly due to HELP apheresis (Figure 4).

**Figure 5:**
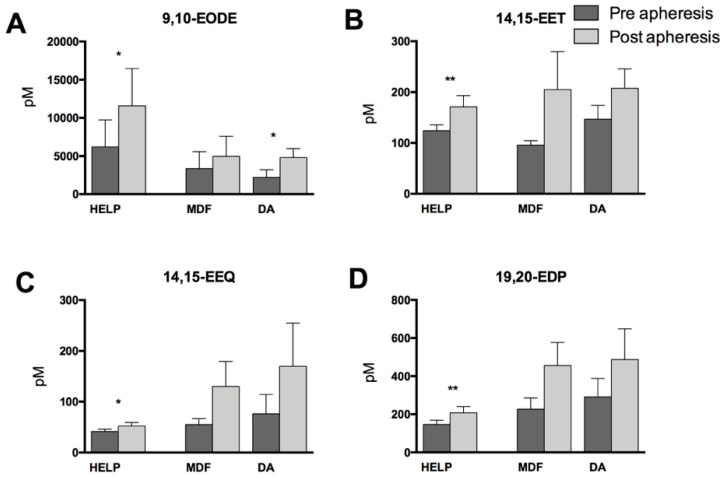
Cytochrome P450 (CYP450)-derived epoxy lipid mediators measured before and after apheresis therapy. (**A**) LA-derived 9,10-epoxyoctadecenoic acid (9,10-EODE); (**B**) AA-derived 14,15-epoxyeicosatrienoic acid (14,15-EET); (**C**) EPA-derived 14,15-epoxyeicosatetraentaenoic acid (14,15-EEQ); and (**D**) DHA-derived 19,20-epoxydocosapentaenoic acid (19,20-EDP). Differences pre- and post-lipoprotein apheresis within one treatment group were significant when indicated (* *p* < 0.05, ** *p* < 0.01). Analysis was performed by two-tailed paired Student’s *t*-test.

Similarly, for a number of CYP-derived epoxy mediators, we found elevated concentrations after lipid apheresis with significant increases in HELP-treated patients for LA-derived 9,10-epoxyoctadecenoic acid (9,10-EODE), AA-derived 14,15-epoxyeicosatrienoic acid (14,15-EET), EPA-derived 14,15-epoxyeicosatetraentaenoic acid (14,15-EEQ), and DHA-derived 19,20-epoxydocosapentaenoic acid (19,20-EDP) (Figure 5). Apheresis using MDF and DA also led to increases of these mediators, but these were not significant, with the exception of 9,10-EODE in DA.

**Figure 6:**
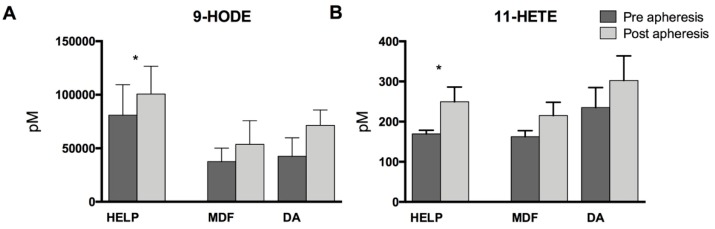
Cyclooxygenase-derived (**A**) linoleic acid (LA, 18:2 *n*-6)-derived 9-hydroxyoctadecadienoic acid (9-HODE) and (**B**) AA-derived 11-hydroxyeicosatetraenoic acid (11-HETE). Analysis was performed by two-tailed paired Student’s *t*-test within the treatment groups. Differences pre- and post-lipoprotein apheresis were significant when indicated (* *p* < 0.05).

Finally, we analyzed cyclooxygenase (COX)-derived prostaglandins and monohydroxy metabolites. While there were no detectable plasma levels of the assayed prostaglandins (data not shown), COX-derived 9-hydroxyoctadecadienoic acid (9-HODE) and 11-hydroxyeicosatetraenoic acid (11-HETE) increased significantly due to apheresis in the HELP treatment group, with non-significant increases in the MDF and DA groups. This might indicate apheresis-triggered COX activation (Figure 6).

## 4. Discussion

This study addressed the changes in plasma lipid mediators derived from omega-6 and omega-3 fatty acids and analyzed them in patients before and after undergoing one of three different lipoprotein apheresis methods (HELP, MFD, and DA). Omega-6 and omega-3 PUFA are important precursors for a broad variety of lipid mediators, which act as mediators in the context of inflammation and cardiovascular diseases [25,26,27].

PUFAs in the blood plasma are either bound as free fatty acids (FFAs) to albumin or are present as part of the TG fraction as phospholipids and cholesterol esters of lipoproteins [28,29]. Recently, we were able to show that PUFA concentrations in plasma are lowered by HELP and DA lipoprotein apheresis methods employed to lower LDL cholesterol in high-risk patients, but not by MDF apheresis [21]. Due to this effect, lipid apheresis might also interfere with the formation of PUFA-derived lipid mediators.

While we were able to confirm our previous data demonstrating lower PUFA amounts in plasma due to HELP and DA lipid apheresis, here we showed a distinct increase of almost all assayed lipid mediators and metabolites post apheresis treatment. Most apparent changes were found in the formation of the 15-LOX metabolites derived from AA, EPA, and DHA as indicated by significantly increased concentrations of 15-HETE, 15-HEPE, and 17-HDHA, particularly in MDF apheresis but also in HELP and DA. Given that these compounds are associated with anti-inflammatory activity [27], this might support the beneficial effect of apheresis treatments, which might even be supported by *n*-3 PUFA supplementation. Lipoprotein apheresis treatment was also associated with an activation of 15-LOX as indicated by increased concentrations of 15-HETE, 15-HEPE, and 17-HDHA. These 15-LOX products have also been associated with the formation of more complex anti-inflammatory mediators: AA-derived 15-HETE can be converted into lipoxin A4 [30], and *n*-3 DHA-derived 17-hydroxydocosahexaenoic acid (17-HDHA) is known to be a precursor of anti-inflammatory D-resolvins [27].

In contrast, the formation of 12-LOX metabolites did not significantly increase and remained nearly constant, particularly in HELP apheresis. This finding is in contrast to our hypothesis based on our pilot study [19]. Since 12-LOX is predominately produced by human platelets, this argues against platelet activation due to HELP apheresis. However, while metabolites of the 12-LOX pathway were only changed moderately by HELP apheresis, MDF and DA induced a much greater increase of 12-LOX metabolites—although these findings were not significant (probably due to the small sample number). Different apheresis methods might thus affect platelet activation differently. In HELP apheresis, plasma is mixed with an acetate-acetic acid buffer (pH at 4.85–5.15) and heparin. Heparin binds to LDL-C, VLDL, and Lp(a), and together with fibrinogen forms insoluble precipitates which are then removed by a polycarbonate membrane. Transient platelet activation accompanied by increased formation of 12-HETE due to heparin treatment has been shown previously [31]. In contrast, cascade filtration or membrane differential filtration (MDF) employs a membrane that effectively retains particles bigger than 1000 kD through which the plasma is circulated after separation of the cellular parts of the blood. While HELP and MDF are plasma-based separation systems, whole blood circulates through a polyacrylate-coated polyacrylamide adsorber in a low-density lipoprotein hemoperfusion and direct absorption of lipoproteins system (DA) [32]. For these whole blood apheresis systems, the polymer surface roughness has been shown to activate thrombocyte adhesion and cellular activation [33]. This may be an explanation for the increased 12-LOX activation in DA compared to HELP but does not explain the effect in MDF. AA-derived 12-HETE has been associated with vasoconstrictive properties in small renal arteries, contributing to arterial hypertension. Furthermore, induction of chemotaxis, neutrophil aggregation, and angiogenesis have been implicated as effects of 12-HETE [20,34]. In connection to diabetes and hyperlipidemia, 12-LOX activation and the formation of 12-HETE have also been shown to induce oxidative stress as well as LDL oxidation [34]. In contrast to *n*-6 AA-derived 12-HETE, the generation of *n*-3 DHA-derived 14-HDHA might confer protective effects, as 14-HDHA is known as a precursor of anti-inflammatory mediators [35,36].

An argument for a pro-inflammatory effect of apheresis treatments, however, is the increased formation of COX-derived 9-HODE and 11-HETE—an effect seen even though almost all patients in this study received low-dose acetylsalicylic acid as part of their routine medication. The effect on COX metabolites is probably not clinically significant, however, as we were not able to detect prostaglandins in the blood plasma assayed in this study.

Besides the lipid mediators derived from cyclooxygenase and the different lipoxygenases, cytochrome P450 (CYP450)-derived lipid mediators increased due to apheresis treatments. AA is also converted by CYP450 enzymes into epoxy-metabolites (EETs) [37]. Multiple beneficial effects have been reported for the family of EETs in the context of cardiovascular diseases. For example, EETs have been shown to cause vasodilatation and to inhibit the activation of platelets [38,39]. EETs are not stable and will be rapidly metabolized into their less-active downstream products, dihydroxyeicosatrienoic acids (DHETs), by the soluble epoxide hydrolase (sEH). Inhibition of sEH has been shown to increase levels of HDL-C and to reduce LDL-C in mice, resulting in a reduction of atherosclerotic plaques [40]. Interestingly, the present study identified an increase of EETs as well as of *n*-3 PUFA-derived epoxy-metabolites 14,15-EEQ and 19,20-EDP, which have been implicated in cardioprotection [18].

Interestingly, HELP apheresis in particular led to higher levels of LA metabolites already at baseline. This observation warrants further study, as it might indicate a sustained activation of LA metabolite formation in patients undergoing regular HELP apheresis treatments, but not in those undergoing MDF or DA treatments.

A limitation of this study is the small number of individuals in each treatment group, which might affect the validity of our data. Nevertheless, we believe that particularly due to our focus on the larger group of HELP apheresis patients, our data are valid and meaningful. However, given the small sample number, we chose not to perform corrections for multiple testing, which is a limitation of this study. The present study does not answer questions regarding long-term effects of different and repeated apheresis treatments on PUFAs and lipid mediators, and did not account for differences in dietary supplementation of *n*-3 and *n*-6 PUFAs specifically in the context of regular apheresis treatments and subsequent changes in lipoprotein levels. Future studies will have to address these questions as well as to determine lipid metabolites in the specific lipoprotein fractions (e.g. VLDL, LDL, HDL).

## 5. Conclusions

In summary, we found that lipoprotein apheresis treatment led to an increased formation of lipid mediators derived from essential omega-6 and omega-3 polyunsaturated fatty acids. This was in spite of the PUFA-lowering effect of these treatments. Given that several of the identified lipid mediators, particularly from omega-3 fatty acids, have been implicated in inflammation-dampening and cardioprotection, we hypothesize that supplementing these fatty acids in apheresis patients could increase the formation of protective lipid mediators in the context of apheresis treatments.

## Figures and Tables

**Table 1:** Patient characteristics. BMI: body mass index; DA: direct absorption of lipoproteins—DALI^®^; HELP: heparin-induced extracorporeal low-density lipoprotein precipitation; MDF: membrane differential filtration.

	Summary/Average	HELP	MDF	DA
**n**	**33**	**17**	**9**	**7**
Age (years)	58.3	57.6	58.4	63.6
Male:Female	30:3	15:2	9:0	6:1
BMI (kg/m^2^)	29.2	27.82	30.5	29.9
**Apheresis indication**				
Hypercholesterolemia	25	12/17	7/9	6/7
Hyperlipoproteinemia (a)	23	14/17	7/9	2/7
**Cardiovascular disease**				
Arterial hypertension	25	13/17	7/9	5/7
CHD	31	17/17	9/9	6/7
condition after myocardial infarction	18	9/17	6/9	3/7
PTCA with stenting	25	15/17	8/9	2/7
CABG	10	4/17	2/9	4/7
Diabetes mellitus type II	3	2/17	-	1/7
**Medication**				
ASS 100	32	17/17	8/9	7/7
Thienopyridine	11	5/17	5/9	1/7
Omega-3 ethylester	18	6/17	8/9	4/7
Statins	27	15/17	8/9	4/7
Ezetimibe	13	6/17	2/9	5/7
ACE inhibitor	12	6/17	5/9	1/7
AT1-blocker	10	6/17	3/9	1/7
ß-blocker	24	12/17	6/9	6/7
Calcium blocker	10	5/17	2/9	3/7
Diuretic	8	3/17	2/9	3/7

**Table 2:** Concentrations and standard deviations of plasma polyunsaturated fatty acids in patients undergoing HELP, MDF, or DA apheresis. (*n* = 17 for HELP, *n* = 9 for MDF, and *n* = 7 for direct adsorption (DA)). Differences between the groups were only significant when indicated (* *p* < 0.05, ** *p* < 0.01, *** *p* < 0.001). Analysis was performed by two-tailed paired Student’s *t*-test.

FA Group	HELP			MDF			DA		
	pre	post		pre	post		pre	post	
	conc. mg/mL	conc. mg/mL	% change	conc. mg/mL	conc. mg/mL	% change	conc. mg/mL	conc. mg/mL	% change
**n-3 FA**									
C18:3 *n*-3	26.04 ± 13.43	16.74 ** ± 7.13	−35.71	23.03 ± 12.28	14.53 ± 5.11	−36.9	41.01 ± 29.30	24.57 ± 15.96	−40.1
C20:5 *n*-3	39.99 ± 16.96	28.79 ** ± 16.80	−28.01	57.21 ± 35.47	48.98 ± 30.00	−14.4	66.12 ± 41.47	37.87 * ± 23.15	42.7
C22:5 *n*-3	18.54 ± 7.22	13.09 *** ± 5.12	−29.38	17.27 ± 7.18	15.59 ± 6.63	−9.8	23.94 ± 8.34	14.98 ± 4.04	−37.4
C22:6 *n*-3	61.26 ± 15.25	45.93 *** ± 18.20	−25.02	60.49 ± 27.80	56.93 ± 27.76	−5.9	92.14 ± 41.66	60.86 ** ± 26.71	−33.9
**n-6 FA**									
C18:2 *n*-6	713.11 ± 209.94	488.11 *** ± 150.23	−31.55	515.50 ± 195.36	430.18 ± 183.21	−16.6	903.77 ± 338.23	508.98 ** ± 189.83	−43.7
C18:3 *n*-6	12.29 ± 7.17	7.25 *** ± 4.22	−41.01	7.84 ± 4.23	7.03 ± 4.66	−10.4	15.76 ± 7.64	8.15 ** ± 4.32	−48.3
C20:2 *n*-6	6.18 ± 2.62	4.53 ** ± 2.50	−26.74	4.20 ± 1.40	3.87 ± 2.30	−8.0	7.17 ± 2.54	4.70 ** ± 1.67	−34.4
C20:3 *n*-6	40.57 ± 10.76	30.03 *** ± 9.75	−25.98	28.35 ± 9.57	29.69 ± 17.12	−4.7	52.93 ± 23.88	33.56 *** ± 16.64	−36.6
C20:4 *n*-6	205.57 ± 78.59	151.68 *** ± 64.79	−26.22	143.67 ± 42.91	154.43 ± 91.39	−7.5	259.19 ± 51.58	158.45 *** ± 33.10	−38.9
C22:4 *n*-6	5.31 ± 3.02	3.52 *** ± 2.23	−33.66	2.98 ± 1.28	3.21 ± 2.03	−7.7	5.77 ± 1.75	3.69 ** ± 1.17	−36.7
**MU-FA**									
C14:1 *n*-5	2.83 ± 2.39	1.54 * ± 1.35	−45.63	2.79 ± 1.68	1.90 ± 1.19	−31.88	3.04 ± 2.39	1.68 ** ± 1.87	−44.65
C16:1 *n*-7	76.31 ± 66.30	48.31 ** ± 45.88	−36.69	54.16 ± 27.30	51.94 ± 38.10	−4.09	98.97 ± 70.18	55.16 * ± 40.86	−44.26
C18:1 *n*-9c	822.93 ± 436.22	545.52 ** ± 326.87	−33.71	546.09 ± 192.72	481.01 ± 288.90	−11.92	1077.42 ± 512.09	665.84 * ± 311.13	−38.2
C18:1 *n*-7c	71.11 ± 35.72	47.87 *** ± 28.26	−32.86	48.86 ± 15.11	47.01 ± 27.14	−3.78	90.70 ± 38.51	90.70 ** ± 38.51	−38.29
C20:1 *n*-9	7.63 ± 4.40	5.73 * ± 3.70	−24.94	4.33 ± 1.53	3.89 ± 2.36	−10.04	10.07 ± 4.87	6.74 * ± 3.17	−33.03
C22:1 *n*-9	1.54 ± 1.36	1.65 ± 1.35	6.87	1.44 ± 0.41	1.10 ± 0.70	−23.38	2.38 ± 1.78	2.25 ± 1.48	−5.56
C24:1 *n*-9	27.66 ± 6.11	18.61 *** ± 5.99	−32.71	20.99 ± 5.89	20.28 ± 10.83	−3.41	40.38 ± 10.59	20.60 *** ± 7.23	−48.98
**S-FA**									
C12:0	5.78 ± 5.24	5.49 ± 6.78	−5.05	5.01 ± 3.66	3.93 ± 3.04	−21.51	6.46 ± 2.03	3.23 ** ± 2.95	−50.03
C14:0	43.39 ± 32.30	25.70 * ± 19.34	−40.76	38.11 ± 19.63	27.90 ± 17.41	−26.80	49.70 ± 30.96	27.72 * ± 24.01	−44.22
C16:0	631.74 ± 183.78	446.20 * ± 152.09	−29.37	594.37 ± 223.44	448.72 ± 186.17	−24.51	782.43 ± 115.50	532.66 * ± 92.20	−31.92
C17:0	9.44 ± 3.73	6.27 *** ± 2.42	33.60	7.18 ± 2.69	6.27 ± 2.74	−12.63	10.26 ± 3.64	6.38 ** ± 2.25	37.81
C18:0	245.11 ± 89.13	180.60 *** ± 79.90	−33.71	175.23 ± 64.06	164.04 ± 84.84	−6.39	285.69 ± 88.19	192.91 ** ± 57.01	−32.48
C20:0	6.44 ± 2.13	4.48 *** ± 2.01	−30.40	4.73 ± 1.67	4.35 ± 2.07	−8.01	8.86 ± 3.05	4.97 ** ± 1.43	−43.89
C22:0	15.44 ± 5.14	10.38 *** ± 4.55	−32.76	11.43 ± 4.19	9.69 ± 4.95	−15.21	23.56 ± 5.92	12.37 *** ± 5.34	−47.51
C24:0	11.13 ± 3.75	7.29 *** ± 2.76	−34.49	8.41 ± 3.45	7.66 ± 3.62	−8.94	16.34 ± 3.81	7.89 *** ± 2.39	−51.7

## Supplementary Materials

The following are available online at http://www.mdpi.com/2072-6643/11/2/363/s1, Figure S1: Polyunsaturated fatty acids in plasma. Table S1: Oxylipins in plasma with standard deviations.
