# Supplementary material for: Activation of Lipid Mediator Formation Due to Lipoprotein Apheresis
Source: Nutrients. 2019 Feb 9;11(2):363. doi: 10.3390/nu11020363 (PMC6412478; doi:10.3390/nu11020363)
Supplement: Supplementary file 1 [file nutrients-11-00363-s001.docx]

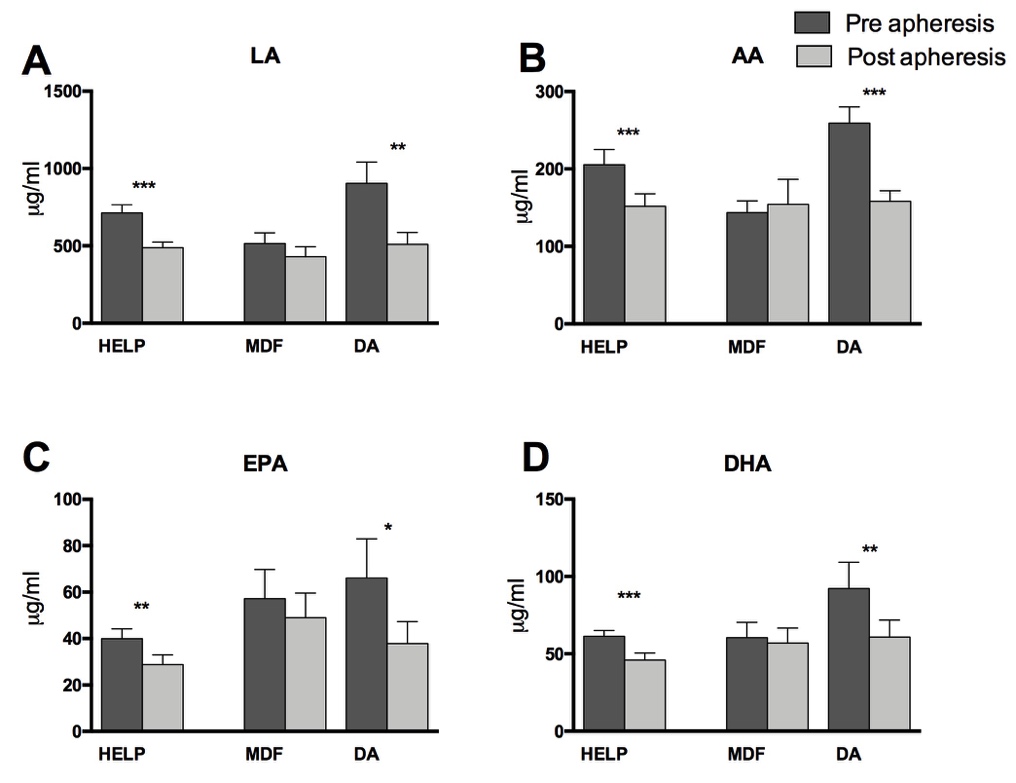


**Figure S1.** Plasma polyunsaturated fatty acids in patients undergoing HELP, MDF, DA apheresis. (A) Linoleic acid (LA, 18:2 n-6), (B) Arachidonic acid (AA, 20:4 n-6), (C) (Eicosapentaenoic acid (EPA, 20:5 n-3), (D) Docosahexaenoic acid (DHA, 22:6 n-3)

**Table S1.** The displayed results show shows concentrations and standard deviations of plasma oxylipins of all patients pre- and post- lipid apheresis therapy (n = 33 for HELP, MDF and DA together) and for each therapy method separately (n= 17 for HELP; n=9 for MDF; n=7 for DA). Differences between the groups were only significant when indicated (* p <0.05, ** p <0.01, *** p<0.001). Analysis was performed by two-tailed paired Student’s t-test.
